# Cardiac Abnormalities in Acromegaly Patients: A Cardiac Magnetic Resonance Study

**DOI:** 10.1155/2020/2018464

**Published:** 2020-02-14

**Authors:** Xiaopeng Guo, Jian Cao, Peijun Liu, Yihan Cao, Xiao Li, Lu Gao, Zihao Wang, Ligang Fang, Zhengyu Jin, Yining Wang, Bing Xing

**Affiliations:** ^1^Department of Neurosurgery, Peking Union Medical College Hospital, Chinese Academy of Medical Sciences and Peking Union Medical College, Beijing 100730, China; ^2^Key Laboratory of Endocrinology, Ministry of Health, Peking Union Medical College Hospital, Chinese Academy of Medical Sciences and Peking Union Medical College, Beijing 100730, China; ^3^China Pituitary Disease Registry Center, Beijing 100730, China; ^4^China Pituitary Adenoma Specialist Council, Beijing 100730, China; ^5^Department of Radiology, Peking Union Medical College Hospital, Chinese Academy of Medical Sciences and Peking Union Medical College, Beijing 100730, China; ^6^Department of Cardiology, Peking Union Medical College Hospital, Chinese Academy of Medical Sciences and Peking Union Medical College, Beijing 100730, China

## Abstract

Cardiac abnormalities are the most common and deadly comorbidities of acromegaly. Assessments using cardiac magnetic resonance (CMR) imaging in acromegaly patients are rare. We aimed to evaluate the frequencies of left ventricular hypertrophy (LVH), interventricular septum hypertrophy (IVSH), LV systolic dysfunction (LVSD), right ventricular systolic dysfunction (RVSD), and myocardial fibrosis (MCF) and detailed quantitative parameters in acromegaly patients using CMR and analyze their correlations with clinical features. Sixty-one patients were enrolled in this study. The rates of LVH, IVSH, LVSD, RVSD, and MCF were 26.2%, 27.9%, 8.2%, 9.8%, and 14.8%, respectively. The average LV mass, LV mass index, IVS thickness, LV and RV free wall thickness, and LV and RV ejection fractions were 114.4 g, 60.0 g/m^2^, 9.6 mm, 7.2 mm, 2.9 mm, 59.9%, and 56.6%, respectively. The LV mass index was larger (68.9 ± 26.0 vs. 48.8 ± 10.6 g/m^2^), the IVS was thicker (10.3 ± 2.8 vs. 8.8 ± 1.8 mm), and the LV (57.6 ± 12.3% vs. 62.8 ± 4.8%) and RV ejection fractions (54.6 ± 8.7% vs. 59.2 ± 5.9%) were lower in male patients than in female patients (all *p* < 0.05). Age, body mass index (BMI), disease duration, and hypertension were associated with cardiac abnormalities (all *p* < 0.05). In conclusion, structural and functional cardiac abnormalities can be comprehensively evaluated by CMR in acromegaly patients. Gender greatly affects the presence of cardiac abnormalities. Age, BMI, disease duration, and hypertension but not GH or IGF-1 levels are associated clinical factors.

## 1. Introduction

Acromegaly is a chronic neuroendocrine disease that is usually caused by pituitary adenoma and characterized by elevated growth hormone (GH) and insulin-like growth factor 1 (IGF-1) levels in serum [1]. Elevated hormone levels and increased cardiac risk factors, including obesity, hypertension, diabetes, and hyperlipemia, first cause and then aggravate heart abnormalities, which are the most common comorbidity observed in acromegaly patients [2–4].

Cardiac comorbidities are responsible for approximately 60% of deaths in acromegaly patients, in whom echocardiography is the method most frequently used to assess changes in cardiac structure and function [4, 5]. The cardiac changes observed in acromegaly patients include left ventricular hypertrophy (LVH), dilated atria and ventricles, and LV diastolic and systolic dysfunction (LVDD and LVSD, respectively) [6, 7].

Cardiac magnetic resonance (CMR) provides advantages over echocardiography, including a higher spatial resolution and the ability to obtain quantitative datasets. CMR is therefore considered the “gold standard” for assessing myocardial mass, heart chamber volume, and ventricular systolic function [8–11]. Studies have also demonstrated that CMR can be used to precisely detect myocardial fibrosis (MCF), ventricular wall thickness, and right ventricular (RV) systolic dysfunction (RVSD) [10, 12]. Given the above advantages, cardiac evaluation by CMR has the potential to detect some secluded or deadly heart diseases that are difficult to detect using echocardiography in acromegaly patients.

Few studies in the literature have used CMR to evaluate cardiac abnormalities in acromegaly patients [13–17]. The reported frequencies of LVH, MCF, and LVSD in acromegaly patients evaluated by CMR are 5–72%, 0–13.5%, and 0–12.5%, respectively. The patient sample sizes included in previous studies have been relatively small (3 of the 5 articles had a sample size of 15 or fewer cases), and the categories of cardiac abnormalities that they evaluated were limited [13–15]. It is therefore difficult to define with relative precision the frequencies and degrees of cardiac structural and functional changes in acromegaly patients based on findings using the gold standard, CMR. Additionally, correlations between cardiac parameters and clinical factors, including patient age, gender, disease duration, body mass index (BMI), disease history, and GH and IGF-1 levels, have not been fully investigated, and the results of previous studies that have explored these correlations remain controversial.

Therefore, the aim of the study was to use CMR to determine the frequencies and degrees of cardiac structural and functional abnormalities in active acromegaly patients in one of China's largest pituitary tumor centers. We also aimed to investigate the influence of clinical indexes and GH and IGF-1 levels on cardiac changes in this patient group.

## 2. Materials and Methods

### 2.1. Patient Population

We prospectively and consecutively enrolled acromegaly patients (age > 18 years) at the Department of Neurosurgery at Peking Union Medical College Hospital (PUMCH) from May 2016 to February 2018. The following inclusion criteria were applied: (1) GH nadir >0.4 ng/ml after a 75 g oral glucose load, random GH > 1 ng/ml, and high plasma IGF-1 level (adopting the age-related normal reference range of IGF-1 level at PUMCH as previously reported [18]) as endocrine indexes [1]; (2) at least one of the typical manifestations of acromegaly [1]; and (3) pituitary adenoma detected on magnetic resonance imaging (MRI) [19]. Patients with the following were excluded: serious hepatic and/or renal dysfunction, claustrophobia, a history of metal implantation, gadopentetic acid allergy, known congenital heart disease, valvular heart disease, or coronary heart disease.

This study was carried out in accordance with the tenets of the Helsinki declaration and was approved by the Institutional Review Board at PUMCH, Chinese Academy of Medical Sciences (Ethical Number: ZS-1293). Each patient signed an informed consent document before enrollment. The protocol was approved by PUMCH, Chinese Academy of Medical Sciences.

### 2.2. Study Protocol

Hormone measurement, contrast-enhanced MRI for sella turcica, CMR, echocardiography, and other routine preoperative examinations were performed in all enrolled patients. The patients' gender, age, BMI, disease duration (months), heart rate (beats/min), arterial blood pressure (mmHg), and history of hypertension, diabetes, hyperlipemia, and smoking were recorded in detail. The levels of GH (ng/ml) and IGF-1 (ng/ml) and the GH nadir (ng/ml) after oral glucose administration were documented. The disease duration was defined as the interval from the onset of typical acromegalic presentations to a definite endocrine diagnosis. Smokers were defined as patients who smoked at least one cigarette per day for at least 6 months. At the time of inclusion, all the patients with hypertension, diabetes, and hyperlipemia were using certain medication, and they were all properly treated with medication during the perioperative period.

### 2.3. Biochemical Assessments and Pituitary MRI

Blood samples were collected at 06 : 00 at the PUMCH Neurosurgery ward after an at least eight-hour fasting period. Chemiluminescence assays (Siemens Healthcare Diagnostics Products Ltd., UK) were used to measure fasting GH and IGF-1 levels. GH levels were also measured at 30 min, 60 min, 120 min, and 180 min after the administration of 75 g of oral glucose.

Contrast-enhanced MRI (Discovery MR750, GE, USA) of the sella turcica was performed in all acromegaly patients. Typical radiological imaging of a pituitary adenoma included a solid hypo/isointense mass on T1WI, a hyper/isointense mass on T2WI, and reduced reinforcement after gadopentetic acid administration. In patients, pituitary adenomas were classified into the following two subtypes according to the largest diameter of the tumor on MRI: microadenoma (≤10 mm) and macroadenoma (>10 mm). Tumor invasiveness was classified according to the Knosp classification system [20], and a radiological presentation of Knosp >2 indicated that the tumor was invasive.

### 2.4. Cardiac Magnetic Resonance Technique

#### 2.4.1. Imaging Equipment and Patient Preparation

CMR was performed in patients on a 3.0 T superconducting whole-body MR scanner (MAGNETOM Skyra, Siemens Healthineers, Germany) with a gradient field of 45 mT/m and a slew rate of 200 T/m/s. An 18-element body matrix coil and a 32-element spine array coil were used for data acquisition. Respiratory gating and electrocardiograph gating were both MRI-compatible, and a wireless vector gating panel was used for electrocardiograph gating.

Before each examination, we required the patient to practice breathing. This practice included end-expiratory breath-holding for longer than 20 s and regular and quiet breathing, which ensured subtle and uninfluential chest movements.

#### 2.4.2. CMR Sequencing

The CMR consisted of the following four parts: (1) localization scanning on the axial, sagittal, and coronal views for chest imaging; (2) fast spin-echo sequence-based cardiac imaging on the long and short axes; (3) cardiac cine images; and (4) contrast-enhanced scanning.

The cine images were acquired with an electrocardiogram-gated 2D balanced steady-state free precession sequence during multiple breath holds. Two-, three- and four-chamber long-axis and 9–11 short-axis slices covering the LV were acquired. The key parameters were as follows: repetition time (TR)/echo time (TE), 3.3/1.43 ms; flip angle (FA), 55°–70°; voxel size, 1.6 × 1.6 × 6.0 mm; temporal resolution, 45.6 ms; bandwidth, 962 Hz/pixel. The other parameters included TR/TE/flip angle, 2.7 ms/1.12 ms/20°; voxel size, 1.4 × 1.4 × 8.0 mm.

A bolus of gadobenate dimeglumine (0.5 mmol/ml, Injection Dimeglumine Gadopentetate, Beijing Beilu Pharmaceutical Co., Ltd.) was given at a dose of 0.05 mmol/kg and a flow rate of 4 ml/s for first pass perfusion imaging. Another bolus of gadobenate dimeglumine at a dose of 0.1 mmol/kg and a flow rate of 1 ml/s was then given, followed by LGE imaging 10–15 min later. LGE images were acquired with a 2D phase-sensitive inversion-recovery (PSIR) gradient-echo pulse sequence. The same long- and short-axis slice positions as for cine images were acquired. The parameters of the sequence were as follows: TR/TE/FA, 5.2 ms/1.96 ms/20°; voxel size, 1.4 × 1.4 × 8.0 mm.

### 2.5. Imaging Analysis

Imaging results were directly transmitted into a postprocessing workstation (Circle Cardiovascular Imaging, version 5.3, Canada). Measurements were performed by two experienced radiologists (J. C. and P. L.) who were blind to the clinical data of the patients. They first reached an agreement regarding their evaluations of the heart layer and cardiac cycle phase. The data presented in this study is the average value of the data evaluated by the two radiologists. Discrepancies were resolved via consensus during a joint evaluation with a third radiologist (Y. W.).

The following parameters were measured: LV mass (LVM), LVM index (LVMi), interventricular septal (IVS) thickness (IVST), LV free wall (LVFW) thickness, RV free wall (RVFW) thickness, LV ejection fraction (LVEF), and RV ejection fraction (RVEF). Late gadolinium enhancement (LGE) was monitored after gadopentetic acid infusion. The frequencies of LVH, IVS hypertrophy (IVSH), LVSD, RVSD, and MCF were calculated based on the CMR reference ranges for the Chinese general population [21]. LVH, IVSH, LVSD, RVSD, and MCF were qualitatively evaluated; and LVM, LVMi, IVST, LVFW, RVFW, LVEF, and RVEF were quantitatively evaluated. MCF was defined as the late gadolinium enhancement on the CMR images.

### 2.6. Echocardiography

All the patients underwent echocardiography with an ultrasound scanner (Vivid E9, GE, Norway) at the PUMCH Cardiology department. Standard parameters related to cardiac structure and function were recorded, including LVEF, IVST, LVFW, LV fractional shortening, LV end-diastolic diameter, and LV end-systolic diameter. Qualitative indexes for LVH, IVSH, and LVSD were also calculated.

### 2.7. Statistical Analysis

SPSS software (SPSS Inc., version 17.0, USA) was used to analyze the data and perform all statistical analyses. GraphPad Prism software (GraphPad Inc., version 5, USA) was used to perform linear correlations. The results are shown as the mean ± standard deviation, numbers or percentages. The *χ*^2^ test was used to analyze categorical variable correlations. Levene's test was used to evaluate the distribution of continuous data. Student's *t*-test or the Mann-Whitney *U* test was used for continuous data comparisons according to the data distribution. A bivariate logistic regression analysis was used to explore independent risk factors for developing cardiac abnormalities in acromegaly patients. A linear correlation analysis was carried out to demonstrate correlations between CMR parameters and clinical data. The *r*^2^ revealed the fitness of the linear correlation analysis with the actual results. Statistical significance was defined as *p* < 0.05.

## 3. Results

### 3.1. General Information

We enrolled 61 Chinese acromegaly patients, including 34 males (56%) and 27 females (44%). The clinical data and hormone levels of the included acromegaly patients are presented in Table 1. Only one patient had a history of transsphenoidal pituitary tumor resection before admission, and the relevant patient was admitted into our hospital this time because of tumor recurrence and nonremission of GH and IGF-1. All other patients were untreated, initially diagnosed as acromegaly patients without a history of surgery, radiation, or somatostatin medication.

BMI was higher in male patients than in female patients (*p*=0.001), as well as IGF-1 levels (*p*=0.004) and the proportion of smokers (*p*=0.002). No significant differences were observed in other clinical indexes and hormone levels between males and females.

Among the patients, 2 had hypogonadism, 3 had hypothyroidism, and 2 had adrenal insufficiency. Relevant hormone replacement was performed in these patients. On MRI, 43 patients (70.5%) were diagnosed with pituitary macroadenomas, and the average largest tumor diameter was 2.5 cm. An invasive pituitary tumor was detected in 21 of the acromegaly patients (34.4%).

### 3.2. CMR Parameters in Acromegaly Patients and Correlation Analysis

**Figure 1:**
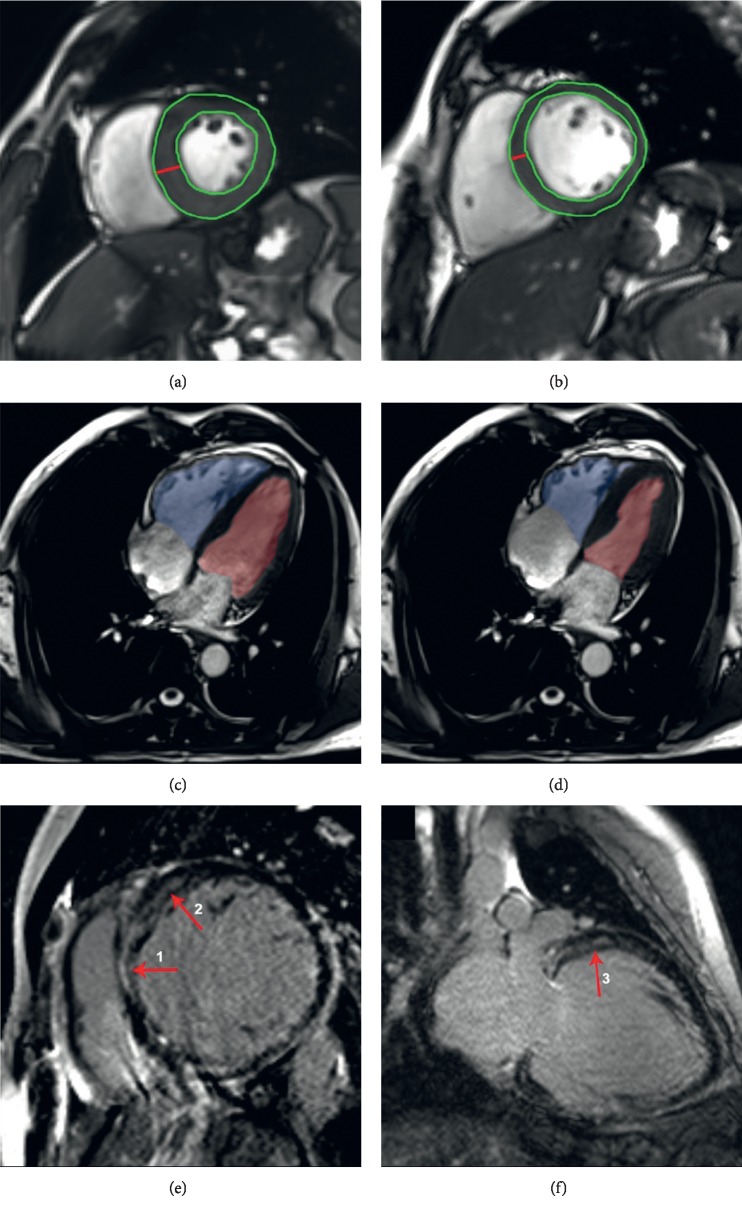
Typical cardiac abnormalities in acromegaly patients evaluated by cardiac magnetic resonance imaging. (a) Interventricular septum (IVS) hypertrophy and left ventricular (LV) hypertrophy shown. (b) Normal IVS thickness and LV mass index. The red lines shown in (a) and (b) indicate the lines along which IVS thickness was measured, and the areas surrounded by green lines reveal the LV mass used for calculations. (c) LV and right ventricular (RV) systolic dysfunction at end-diastolic stage. (d) LV and RV systolic dysfunction at end-systolic stage. The images shown in (c) and (d) were recorded from the same patient. The red area represents the LV volume, and the blue areas represent the RV volume. (e) Mid-wall late gadolinium enhancement (LGE) of the IVS myocardium (red arrow “1”) and the LV myocardium on the cardiac short axis (red arrow “2”). (f) Mid-wall LGE of the basal anterior LV myocardium on the cardiac long axis (red arrow “3”).

The frequencies of cardiac abnormalities and quantitative parameters are summarized in Table 2. A correlation analysis between cardiac comorbidity occurrence and clinical indexes and hormone levels is presented in Table 3. A linear correlation analysis between cardiac quantitative parameters and clinical factors is shown in Table 4. Typical CMR images of cardiac abnormalities observed in the included acromegaly patients are shown in Figure 1.

#### 3.2.1. Ventricular Hypertrophy

In this study, the average LVM (*p* < 0.001) and LVMi (*p* < 0.001) were significantly higher in male patients than in female patients. BMI was higher (*p*=0.011), the disease duration was longer (*p*=0.036), and the frequency of hypertension was higher (*p*=0.048) in patients with LVH than in those without LVH. LVMi values were positively correlated with BMI (*p* < 0.05). LVH in all patients was asymmetric. The LVH cases in most of the patients were concentric (93.8%, 15/16), and only 1 was centrifugal.

The proportion of patients with IVSH was significantly higher in male patients (38.2%) than in female patients (14.8%). In all, 10 of the 17 patients with IVSH were diagnosed with LVH at the same time (16.4% of all patients). BMI was higher (*p*=0.010), the patients were older (*p*=0.013), and the proportion of males was higher (*p*=0.043) in the patients with IVSH than in those without. The independent risk factors for developing IVSH were older age (*p*=0.003) and male gender (*p*=0.028).

The average IVST was 9.6 ± 2.5 mm, and the interventricular septum was thicker in male patients than in female patients (*p* < 0.001). IVST was positively correlated with patient age, BMI, and disease duration (*p* < 0.05). Additionally, the interventricular septum was thicker in patients with hypertension than in those without (*p*=0.009). In these patients, IVST was also positively and linearly correlated with LVM and LVMi (*p* < 0.05).

The LVFW was thicker than the RVFW in acromegaly patients (*p* < 0.001). In addition, The LVFW (*p*=0.004) and RVFW (*p*=0.010) were both thicker in male than in female acromegaly patients. The thickness of the LVFW was positively correlated with patient BMI.

#### 3.2.2. Left and Right Ventricular Systolic Function

LVSD was detected in 5 patients (8.2%), 3 of whom had an LVEF higher than 40%. Two patients were diagnosed with severe LVSD and exhibited remarkably decreased LVEF (13.7% and 21.3%). All 5 patients with LVSD were males, of whom 2 were also diagnosed with LVH and IVSH. Acromegaly patients with LVSD were older (*p* < 0.005) and were more likely than those without LVSD to have hypertension (*p*=0.034). The average LVEF in the included acromegaly patients was 59.9% ± 10.0%, and it was 5.2% lower in male patients than in female patients (*p*=0.031). LVEF was negatively and linearly correlated with both LVM and LVMi.

Among the 6 patients with RVSD, 4 had an RVEF higher than 40%, and 2 had an RVEF between 30 and 40%. In all, 3 of the 6 patients with RVSD also developed LVSD. Compared to patients without RVSD, the patients with RVSD had higher BMI (*p*=0.007) and longer disease duration (*p*=0.003) and were more likely to have diabetes mellitus (*p*=0.025). The average RVEF in these acromegaly patients was 56.6% ± 7.9% and was 4.6% lower in male patients than in female patients (*p*=0.021). RVEF was negatively correlated with BMI. Additionally, RVEF was lower in patients with diabetes mellitus than in those without (*p*=0.004). RVEF was positively and linearly correlated with LVEF in acromegaly patients.

#### 3.2.3. Myocardial Fibrosis

LGE, which indicates the presence of MCF, was detected on CMR in 9 patients (14.8%). The focal fibroses in 8 patients were located in the middle layer of the myocardium, and transmural fibrosis was found in 1 patient. The heart segments involved included the LV (4/61, 6.6%), the interventricular septum (1/61, 1.6%) and both (4/61, 6.6%). Among patients with MCF, 6 had LVH and IVSH, 3 had LVSD, and 4 had RVSD.

Compared with patients without MCF, the patients with MCF had higher BMI (*p*=0.011) and were more likely to be males (*p*=0.030). A bivariate logistic regression analysis revealed that the male gender was an independent risk factor for MCF in acromegaly patients (*p*=0.026).

### 3.3. Correlations between CMR and Echocardiography Findings

The echocardiography data are summarized in Supplemental [Supplementary-material supplementary-material-1]. Five patients had LVH (8.2%), and 9 patients had IVSH (14.8%). Two patients were diagnosed with LVSD (LVEF = 27% and 34%) and also had LVH and IVSH. RVEF and MCF cannot be precisely assessed by echocardiography. The LVEF was 64.7% ± 8.5% and was lower in male than in female patients (62.7% ± 9.9% versus 67.2% ± 5.4%, *p*=0.039).

LVEF (*r*^2^ = 0.354, *p* < 0.001), IVST (*r*^2^ = 0.216, *p* < 0.001), and the thickness of the LVFW (*r*^2^ = 0.121, *p* < 0.006) were evaluated by echocardiography and positively correlated with the findings observed on CMR.

## 4. Discussion

In this study, we used CMR to investigate the frequencies of cardiac abnormalities and detailed quantitative cardiac parameters in acromegaly patients and then analyzed the correlations between the changes in cardiac structure and function and patients' clinical indexes and GH and IGF-1 levels. This study comprehensively and systematically evaluated the cardiac comorbidities by CMR and related clinical factors in acromegaly patients. To the best of our knowledge, the included sample population is the first Asian population and the largest reported so far in the literature.

Five previous studies have used the gold standard, CMR, to evaluate cardiac abnormalities in acromegaly patients (Table 5) [13–17]. The 2010 study by Bogazzi et al. [22] is not included in Table 5 because the patient sample was the same as that included in the study conducted by the same authors in 2008 [13]. The CMR results for acromegaly patients varied among different studies. The 2008 study by Bogazzi et al. [13] was the first study to demonstrate that the frequency of LVH in acromegaly patients was 72% as evaluated by CMR. This frequency was much higher than 36% as detected by echocardiography in the same group, and the frequencies of MCF and LVSD were both 0% [13]. However, recent studies by Silva et al. [16] and Warszawski et al. [17] have shown that the frequency of LVH in acromegaly patients is 5–8% and that MCF is present in 12–13.5% of these patients. Although the exact frequencies of different cardiac abnormalities remain controversial, this study and previous ones provide a foundation for applying CMR to evaluate cardiac involvement in acromegaly patients.

In this study, the proportion of acromegaly patients with LVH was 26.2%, which is within the range of LVH frequencies reported in the literature (5–72%). The average LVMi, a quantitative index of LVH, was 77–92 g/m^2^ in studies published in European countries [13–15] but only 61.7-61.8 g/m^2^ in studies published in Brazil [16, 17]. Our results produced an average LVMi of 60.0 g/m^2^, which is very close to the results of studies originating in Brazil but much lower than those reported in European studies. One possible explanation for these reported differences in LVMi is the degree of development (i.e., undeveloped versus developed countries) and the ethnicities of the enrolled patients (e.g., Asian versus Caucasian). Studies including patients from less developed countries (e.g., China and Brazil) or that including non-Caucasian populations reported lower LVMi values than those reported in more developed countries and Caucasian populations (European countries).

Previous studies that used CMR have demonstrated that LVMi is not correlated with IGF-1 levels, age, disease duration, or hypertension [13, 16, 17]. However, our results led to a different conclusion. We identified potential clinically correlated factors and found that patients' LVM and LVMi values were significantly affected by BMI, disease duration, history of hypertension, and gender. Taking into consideration the sample sizes of previous studies, we chose to use a larger patient sample in this study. This large sample helped us to identify statistically significant correlations that allowed us to successfully detect correlated factors for cardiac involvement in acromegaly patients.

Whether gender has an impact on acromegalic CMR parameters is unknown. We found that quantitative cardiac parameters were significantly different between male and female patients. Gender should be seriously considered as a correlated factor in clinics when evaluating quantitative changes related to cardiac comorbidity. We hypothesized that the differences between male and female patients might influence the occurrence of cardiac abnormalities in this population. By comparing BMI, smoking, IGF-1 levels, and cardiac abnormalities, as shown in Table 4, we found that BMI but not IGF-1 levels and smoking was correlated with quantitative cardiac abnormalities. According to this result, we hypothesized gender might influence cardiac parameters via its impact on BMI.

In this study, the frequency of IVSH based on CMR findings was higher than that found in previous studies, which have reported rates of 0–17.5% [13, 16]. Our results showed that among the 16 patients with LVH and 17 patients with IVSH, 10 had developed both LVH and IVSH. Our results also showed that LVMi and IVST were positively correlated, leading us to hypothesize that LVH and IVSH might share a similar pathogenesis. In addition, our results showed that age, gender, BMI, disease duration, and hypertension were linearly correlated with IVST, in contrast to the results of Bogazzi et al. [13]. The lack of significance in this result might be explained by the small sample size (14 cases) in that study.

Here, we demonstrate for the first time that the frequency of RVSD is 9.8% in acromegaly patients. The proportion of patients with LVSD was within the range of 0–12.5% reported in the literature [13–17]. The significantly positive correlation observed between LVEF and RVEF indicated that ventricular function exhibited synergistic changes in acromegaly patients. The average reported LVEF is 67–68% in patients from European countries [13–15] but only 61.8% in Brazilians [16]. Similarly, the LVEF found in our patients (59.9%) was close to that reported in Brazilians but not to that reported in Europeans. Because LVEF and LVMi were negatively correlated, the degree of development of the study country and the ethnicity of the enrolled patients may also explain the observed differences in LVEF.

It is noteworthy that, in the echocardiography data, the two lowest LVEF values among the patients were 27% and 34%, whereas the CMR findings showed that the LVEF values were 13.7% and 21.3%. The cardiac function evaluation by echocardiography was based on a hypothetical model, whereas the assessment by CMR was based on the quantitative measurement of the dynamic changes of the chambers; the result would even be inaccurate if the cardiac function was lower [21]. Therefore, CMR is more accurate for acromegaly patients with severe heart disease than echocardiography, especially for ventricular systolic function.

By evaluating LGE after gadopentetic acid injection on CMR, we found that fibrosis always developed in the mid-myocardium of the LV or IVS. The proportion of patients with MCF was slightly higher in our patients than the 0–13.5% reported in previous studies [13, 16, 17]. The studies by Silva et al. [16] and Warszawski et al. [17] did not suggest a correlation between MCF and LVH. However, we found that 6 of the 9 patients with MCF had lesions indicating both LVH and IVSH, and 3 of these 9 patients developed LVSD, indicating that myocardial hypertrophy and ventricular systolic dysfunction are to some extent associated with the development of MCF. In addition, among the 6 patients with RVSD, 4 patients (66.7%, 4/6) were diagnosed with MCF (44.4%, 4/9), implying that all these cardiac lesions might have developed synchronously.

In this study, we found that, in the included acromegaly patients, the levels of GH and IGF-1 were not correlated with the occurrence of cardiac abnormalities or quantitative cardiac parameters. In the literature, cardiovascular diseases are generally evaluated in acromegaly patients with echocardiography, and many studies have concluded that having high levels of GH and IGF-1 does not increase the risk of developing LVH or other cardiovascular diseases [7, 23]. However, other parameters, including disease duration, age, BMI, hypertension, and diabetes, have been found to be significantly associated with cardiovascular changes [3, 7, 24–29]. We found that the results of the clinical correlation analysis of the cardiovascular changes reported in previous studies that used echocardiography were in accordance with the results found in our study using CMR.

The correlations reported for LVH and LV systolic function between studies using echocardiography and CMR are in perfect agreement [13, 16, 22]. Therefore, we conclude that both methods are reliable for evaluating cardiac comorbidity in acromegaly patients. However, CMR may more precisely assess MCF and RV systolic function, which cannot be evaluated by the frequently used echocardiography [21]. In addition, CMR is currently recognized as the “gold standard” for evaluating cardiac mass, function, and fibrosis [8–11]. Thus, we recommend that CMR should be considered the first-line examination method for evaluating cardiac structural and functional changes in acromegaly patients.

Some limitations should be mentioned. First, in this study, we enrolled acromegaly patients of only one race, Asian, resulting in inevitable selection bias. Second, because of the low frequency of acromegaly, only 61 cases were included in this study. A multicenter study with a larger sample size and postoperative follow-up to explore the reversibility of cardiac abnormalities is needed.

## 5. Conclusions

CMR can be recommended as a precise and comprehensive examination method to evaluate changes in cardiac structure and function in acromegaly patients. The clinical indexes, but not the hormone levels of GH and IGF-1, were significantly associated with different types of cardiac abnormalities. The LVMi, IVST, LVFW, and RVEF were all correlated with BMI. The IVST was also related to age and disease duration.

## Figures and Tables

**Table 1:** Clinical data and hormone levels of acromegaly patients.

	Whole group (*n* = 61)	Male patients (*n* = 34)	Female patients (*n* = 27)
Age (year)	43.2 ± 13.3	40.6 ± 12.2	46.4 ± 14.0
Body mass index (kg/m^2^)	26.1 ± 3.8	27.5 ± 4.1	24.4 ± 2.6^*∗*^
Disease duration (month)	78.5 ± 57.0	82.4 ± 58.6	73.6 ± 55.7
GH (ng/ml)	36.3 ± 43.7	35.7 ± 44.0	42.6 ± 57.8
GH nadir (ng/ml)	28.2 ± 41.4	26.1 ± 35.3	30.8 ± 48.5
IGF-1 (ng/ml)	896.4 ± 253.9	978.9 ± 234.1	792.6 ± 243.0^*∗*^
Heart rate (beats/min)	78.1 ± 6.1	78.0 ± 6.7	78.2 ± 5.3
Systolic blood pressure (mmHg)	122.6 ± 11.6	121.4 ± 10.5	124.2 ± 12.9
Diastolic blood pressure (mmHg)	77.3 ± 8.9	78.8 ± 9.6	75.5 ± 7.8
Arterial hypertension (yes/no, %)	11/50, 18.0	9/25, 26.5	2/25, 7.4
Hyperlipemia (yes/no, %)	8/53, 13.1	5/29, 8.2	3/24, 4.9
Diabetes mellitus (yes/no, %)	8/53, 13.1	4/30, 6.6	4/23, 6.6
Smoking habit (yes/no, %)	12/49, 19.7	11/23, 32.4	1/26, 3.7^*∗*^

GH, growth hormone; IGF-1, insulin-like growth factor 1. Values are presented as frequencies (%) and the mean ± standard deviation. ^*∗*^It means the difference between male and female patients is significant (*p* < 0.05).

**Table 2:** Cardiac magnetic resonance parameters in acromegaly patients.

Cardiac magnetic resonance parameters	Whole group (*n* = 61)	Male patients (*n* = 34)	Female patients (*n* = 27)
LVM (g)	114.4 ± 51.5	138.6 ± 55.9	84.0 ± 20.8^*∗*^
LVMi (g/m^2^)	60.0 ± 22.8	68.9 ± 26.0	48.8 ± 10.6^*∗*^
LV hypertrophy (yes/no)/(%)	16/45 (26.2)	12/22 (35.3)	4/23 (17.4)
IVST (mm)	9.6 ± 2.5	10.3 ± 2.8	8.8 ± 1.8^*∗*^
Increased IVST (yes/no)/(%)	17/44 (27.9)	13/21 (38.2)	4/23 (17.4)^*∗*^
LVFW (mm)	7.2 ± 1.8	7.8 ± 1.9	6.5 ± 1.5^*∗*^
RVFW (mm)	2.9 ± 1.2	3.2 ± 1.3	2.5 ± 0.9^*∗*^
LV ejection fraction (%)	59.9 ± 10.0	57.6 ± 12.3	62.8 ± 4.8^*∗*^
LV systolic dysfunction (yes/no)/(%)	5/56, (8.2)	5/29 (13.9)	0/27 (0)
RV ejection fraction (%)	56.6 ± 7.9	54.6 ± 8.7	59.2 ± 5.9^*∗*^
RV systolic dysfunction (yes/no)/(%)	6/55 (9.8)	6/28 (17.6)	0/27 (0)
Late gadolinium enhancement (yes/no)/(%)	9/52 (14.8)	8/26 (23.5)	1/26 (3.8)

LVM, left ventricular mass; LVMi, left ventricular mass index; LV, left ventricular; IVST, interventricular septum thickness; LVFW, left ventricular free wall; RVFW, right ventricular free wall; RV, right ventricular. Values are presented as frequencies (%) and the mean ± standard deviation. ^*∗*^It means the difference between male and female patients is significant (*p* < 0.05).

**Table 3:** Risk factors related to myocardial hypertrophy, systolic function and myocardial fibrosis.

	LVH+ (*n* = 16)	LVH− (*n* = 45)	IVSH+ (*n* = 17)	IVSH− (*n* = 44)	LVSD+ (*n* = 5)	LVSD− (*n* = 56)	RVSD+ (*n* = 6)	RVSD− (*n* = 55)	MCF+ (*n* = 9)	MCF− (*n* = 52)
GH (ng/ml)	56.1 ± 72.6	33.0 ± 38.5	47.5 ± 68.0	35.8 ± 41.7	6.0 ± 2.3	42.0 ± 51.3	10.7 ± 8.1	42.2 ± 51.8	52.4 ± 81.8	36.8 ± 43.2
GH nadir (ng/ml)	47.2 ± 65.2	21.4 ± 26.6	40.0 ± 62.3	23.7 ± 29.4	5.0 ± 1.9	30.3 ± 42.6	9.2 ± 7.7	30.3 ± 43.0	44.1 ± 72.6	25.5 ± 33.7
IGF-1 (ng/ml)	887.9 ± 249.0	899.5 ± 258.3	912.7 ± 299.5	890.2 ± 237.5	855.2 ± 420.0	900.1 ± 239.4	963.8 ± 381.5	889.1 ± 240.0	859.0 ± 214.7	902.9 ± 261.3
Age (years)	47.7 ± 12.5	41.2 ± 13.6	50.8 ± 14.4^*∗*^	39.9 ± 12.0	58.0 ± 8.0^*∗*^	41.5 ± 13.0	50.0 ± 10.8	42.2 ± 13.7	48.8 ± 9.9	42.0 ± 14.0
Body mass index (kg/m^2^)	28.2 ± 3.6^*∗*^	25.4 ± 3.6	28.0 ± 3.7^*∗*^	25.4 ± 3.6	28.7 ± 3.1	25.9 ± 3.8	31.3 ± 5.8^*∗*^	25.6 ± 3.1	28.7 ± 3.3^*∗*^	25.7 ± 3.7
Disease duration (months)	102 ± 61.7^*∗*^	70.1 ± 53.6	93.9 ± 50.1	72.5 ± 59.0	81.6 ± 41.0	78.2 ± 58.5	144.0 ± 56.3^*∗*^	71.3 ± 52.8	101.3 ± 72.3	74.5 ± 53.9
Arterial hypertension (yes/no)/(%)	6/10, 37.5%^*∗*^	5/40, 11.1%	6/11, 35.3%	5/39, 11.4%	3/2, 60.0%^*∗*^	8/48, 14.3%	4/2, 66.7%	7/48, 12.7%	4/5, 44.4%	7/45, 13.5%
Diabetes mellitus (yes/no)/(%)	4/12, 25.0%	4/41, 8.9%	4/13, 23.5%	4/40, 9.1%	2/3, 40.0%	6/50, 10.7%	3/3, 50.0%^*∗*^	5/50, 9.1%	1/8, 11.1%	7/45, 13.5%
Hyperlipemia (yes/no)/(%)	1/15, 6.3%	7/38, 15.6%	2/15, 11.8%	6/38, 13.6%	0/5, 0.0%	8/48, 14.3	0/6, 0.0%	8/47, 14.5%	1/8, 11.1%	7/45, 13.5%
Smoking habit (yes/no)/(%)	6/10, 37.5%	6/39, 13.3%	6/11, 35.3%	6/38, 13.6%	2/3, 40.0%	10/46, 17.9%	3/3, 50.0%	9/46, 16.4%	3/6, 33.3%	9/43, 17.3%

GH, growth hormone; IGF-1, insulin-like growth factor 1; LVH, left ventricular hypertrophy; IVSH, interventricular septum hypertrophy; LVSD, left ventricular systolic dysfunction; RVSD, right ventricular systolic dysfunction; MCF, myocardial fibrosis. Values are presented as frequencies (%) and the mean ± standard deviation. ^*∗*^It means the difference between male and female patients is significant (*p* < 0.05).

**Table 4:** Linear correlation analysis between the cardiac magnetic resonance parameters and clinical data.

		LVMi (g/m^2^)	IVST (mm)	LVFW (mm)	RVFW (mm)	LVEF (%)	RVEF (%)
GH (ng/ml)	*r* ^2^	0.000	0.000	0.001	0.001	0.007	0.002
	*p*	0.098	0.928	0.800	0.853	0.508	0.731
GH nadir (ng/ml)	*r* ^2^	0.002	0.003	0.000	0.000	0.005	0.002
	*p*	0.760	0.700	0.886	0.874	0.602	0.736
IGF-1 (ng/ml)	*r* ^2^	0.001	0.001	0.023	0.001	0.001	0.041
	*p*	0.841	0.796	0.244	0.798	0.856	0.116
Age (years)	*r* ^2^	0.031	0.159^a^	0.005	0.015	0.040	0.016
	*p*	0.171	0.002^*∗*^	0.602	0.346	0.125	0.334
Body mass index (kg/m^2^)	*r* ^2^	0.123^a^	0.087^a^	0.111^a^	0.010	0.053	0.106^b^
	*p*	0.006^*∗*^	0.021^*∗*^	0.009^*∗*^	0.433	0.076	0.011^*∗*^
Disease duration (months)	*r* ^2^	0.046	0.108^a^	0.012	0.004	0.028	0.058
	*p*	0.098	0.010^*∗*^	0.400	0.645	0.201	0.062

GH, growth hormone; IGF-1, insulin-like growth factor 1; LVMi, left ventricular mass index; IVST, interventricular septum thickness; LVFW, left ventricular free wall; RVFW, right ventricular free wall; LVEF, left ventricular ejection fraction; RVEF, right ventricular ejection fraction; ECV, extracellular volume. ^*∗*^This indicates that the linear correlation between the CMRi parameter and the clinical data is significant (*p* < 0.05). ^a^ indicates a positive linear correlation. ^b^ indicates a negative linear correlation.

**Table 5:** Literature review of the studies that applied CMRi in acromegaly patients.

Year	First author	Patients enrolled	LVH (%)	LVMi (g/m^2^)	IVSH (%)	LVSD (%)	LVEF (%)	RVSD (%)	MCF (%)
2008	Bogazzi F.	14	72	77 ± 9	0	0	67 ± 11	NA	0
2008	Gouya H.	15	27	77 ± 5.3	NA	0	68 ± 6.6	NA	NA
2010	Andreassen M.	8	37.5	92	NA	12.5	67	NA	NA
2015	Silva C. M.	40	5	61.7 ± 18.8	17.5	0	61.8 ± 9.2	NA	13.5
2016	Warszawski L.	36	8	61.8 ± 19.6	NA	0	NA	NA	12
2018	Present study	61	26.2	60.0 ± 22.8	27.9	8.2	59.9 ± 10.0	9.8	14.8

CMRi, cardiac magnetic resonance imaging; LVH, left ventricular hypertrophy; LVMi, left ventricular mass index; IVSH, interventricular septum hypertrophy; LVSD, left ventricular systolic dysfunction; LVEF, left ventricular ejection fraction; RVSD, right ventricular systolic dysfunction; MCF, myocardial fibrosis; NA, not available.

## Data Availability

The data used to support the findings of this study are included within the article.

## Abbreviations

BMI: Body mass index
CMR: Cardiac magnetic resonance
GH: Growth hormone
IGF-1: Insulin-like growth factor 1
IVSH: Interventricular septum hypertrophy
IVST: Interventricular septum thickness
LV: Left ventricular
LVEF: Left ventricular ejection fraction
LVFW: Left ventricular free wall
LVH: Left ventricular hypertrophy
LVM: Left ventricular mass
LVMi: Left ventricular mass index
LVSD: Left ventricular systolic dysfunction
MCF: Myocardial fibrosis
RV: Right ventricular
RVEF: Right ventricular ejection fraction
RVFW: Right ventricular free wall
RVSD: Right ventricular systolic dysfunction.

## Supplementary Materials

Supplementary Materials Supplemental Table 1: echocardiography data of the enrolled acromegaly patients.
