# Supplementary material for: Cardiac Abnormalities in Acromegaly Patients: A Cardiac Magnetic Resonance Study
Source: Int J Endocrinol. 2020 Feb 14;2020:2018464. doi: 10.1155/2020/2018464 (PMC7042537; doi:10.1155/2020/2018464)
Supplement: Supplementary Materials — Supplemental Table 1: echocardiography data of the enrolled acromegaly patients. [file 2018464.f1.docx]

Supplemental Table 1: Echocardiography data of the enrolled acromegaly patients.

| NO. | ID | Gender | Age | LVEF (%) | IVST (mm) | LVFW (mm) | LVFS (%) | LV end-diastolic diameter (mm) | | LV end-systolic diameter (mm) |
| --- | --- | --- | --- | --- | --- | --- | --- | --- | --- | --- |
| 1 | 42598012 | F | 52 | 61 | 7 | 7 | 33 | 52 | 35 | |
| 2 | 42634462 | M | 50 | 27 | 11 | 11 | 13 | 94 | 82 | |
| 3 | 42674911 | F | 44 | 72 | 9 | 10 | 42 | 55 | 32 | |
| 4 | 42689666 | M | 66 | 63 | 10 | 9 | 35 | 54 | 35 | |
| 5 | 42631398 | F | 58 | 59 | 8 | 8 | 31 | 44 | 30 | |
| 6 | 42705917 | M | 67 | 34 | 9 | 8 | 16 | 68 | 57 | |
| 7 | 42618161 | M | 43 | 68 | 8 | 10 | 38 | 52 | 32 | |
| 8 | 42632418 | M | 27 | 68 | 10 | 10 | 39 | 54 | 33 | |
| 9 | 42734724 | F | 73 | 68 | 9 | 9 | 38 | 55 | 34 | |
| 10 | 42716321 | M | 52 | 72 | 8 | 10 | 42 | 55 | 32 | |
| 11 | 42755927 | F | 63 | 71 | 10 | 10 | 41 | 51 | 30 | |
| 12 | 42741281 | M | 22 | 75 | 8 | 8 | 44 | 50 | 28 | |
| 13 | 42853804 | F | 22 | 62 | 7 | 7 | 33 | 44 | 30 | |
| 14 | 42859206 | F | 39 | 72 | 9 | 8 | 41 | 49 | 29 | |
| 15 | 42851899 | M | 18 | 67 | 8 | 8 | 37 | 49 | 31 | |
| 16 | 42897037 | M | 36 | 68 | 9 | 9 | 38 | 49 | 30 | |
| 17 | 42932194 | F | 30 | 69 | 9 | 8 | 38 | 46 | 28 | |
| 18 | 42899061 | M | 51 | 76 | 11 | 11 | 45 | 50 | 27 | |
| 19 | 42946580 | F | 47 | 60 | 7 | 7 | 32 | 46 | 31 | |
| 20 | 41058914 | M | 33 | 68 | 8 | 8 | 38 | 50 | 31 | |
| 21 | 43018281 | M | 30 | 67 | 7 | 7 | 37 | 56 | 35 | |
| 22 | 43052392 | M | 35 | 61 | 9 | 9 | 33 | 56 | 37 | |
| 23 | 16202451 | F | 54 | 67 | 9 | 9 | 37 | 46 | 29 | |
| 24 | 42238646 | F | 34 | 72 | 9 | 9 | 41 | 44 | 26 | |
| 25 | 43174382 | M | 32 | 66 | 10 | 10 | 37 | 53 | 34 | |
| 26 | 40799447 | M | 37 | 75 | 9 | 9 | 44 | 54 | 30 | |
| 27 | 43210359 | M | 39 | 64 | 9 | 9 | 35 | 47 | 30 | |
| 28 | 42848901 | F | 43 | 77 | 8 | 8 | 46 | 46 | 25 | |
| 29 | 43214168 | F | 29 | 67 | 7 | 7 | 37 | 43 | 27 | |
| 30 | 43237329 | F | 53 | 74 | 8 | 8 | 44 | 49 | 28 | |
| 31 | 43335259 | M | 36 | 61 | 10 | 10 | 33 | 51 | 34 | |
| 32 | 42162826 | M | 33 | 65 | 9 | 9 | 36 | 50 | 32 | |
| 33 | 43310843 | M | 42 | 66 | 6 | 6 | 36 | 48 | 31 | |
| 34 | 43407734 | F | 51 | 55 | 8 | 8 | 28 | 48 | 34 | |
| 35 | 42803756 | F | 35 | 68 | 7 | 6 | 38 | 47 | 29 | |
| 36 | 43377766 | F | 41 | 66 | 7 | 8 | 37 | 45 | 28 | |
| 37 | 43410366 | M | 33 | 71 | 9 | 9 | 40 | 53 | 31 | |
| 38 | 43385356 | F | 72 | 71 | 10 | 10 | 41 | 54 | 32 | |
| 39 | 43433711 | M | 55 | 66 | 7 | 8 | 36 | 45 | 29 | |
| 40 | 43298206 | F | 55 | 59 | 9 | 8 | 31 | 41 | 28 | |
| 41 | 43280480 | F | 48 | 68 | 7 | 7 | 38 | 47 | 29 | |
| 42 | 43389350 | M | 54 | 70 | 12 | 12 | 41 | 60 | 36 | |
| 43 | 18390265 | F | 71 | 65 | 9 | 9 | 36 | 45 | 29 | |
| 44 | 43578846 | F | 40 | 74 | 11 | 11 | 44 | 54 | 31 | |
| 45 | 43303931 | M | 46 | 62 | 12 | 7 | 33 | 49 | 33 | |
| 46 | 43641305 | F | 49 | 64 | 9 | 9 | 35 | 46 | 30 | |
| 47 | 18008552 | F | 37 | 66 | 7 | 7 | 37 | 55 | 35 | |
| 48 | 40933411 | F | 55 | 64 | 8 | 8 | 36 | 52 | 33 | |
| 49 | 17506976 | F | 34 | 74 | 7 | 7 | 43 | 48 | 27 | |
| 50 | 43767569 | M | 30 | 65 | 7 | 7 | 36 | 49 | 32 | |
| 51 | 43787586 | M | 48 | 62 | 7 | 7 | 34 | 56 | 37 | |
| 52 | 43843165 | M | 37 | 59 | 7 | 8 | 32 | 52 | 36 | |
| 53 | 43764474 | M | 34 | 57 | 9 | 9 | 30 | 57 | 40 | |
| 54 | 43992492 | M | 59 | 58 | 12 | 9 | 31 | 51 | 35 | |
| 55 | 43999421 | M | 60 | 58 | 12 | 10 | 31 | 55 | 38 | |
| 56 | 43039710 | M | 39 | 55 | 9 | 8 | 36 | 51 | 33 | |
| 57 | 43834845 | M | 43 | 61 | 7 | 8 | 37 | 58 | 32 | |
| 58 | 44007595 | M | 42 | 64 | 13 | 12 | 35 | 55 | 35 | |
| 59 | 44131917 | M | 27 | 52 | 9 | 9 | 36 | 50 | 33 | |
| 60 | 44148724 | F | 23 | 69 | 7 | 7 | 39 | 48 | 30 | |
| 61 | 44161000 | M | 26 | 60 | 9 | 9 | 32 | 48 | 33 | |
